# MAIT Cell Activation and Functions

**DOI:** 10.3389/fimmu.2020.01014

**Published:** 2020-05-27

**Authors:** Timothy S. C. Hinks, Xia-Wei Zhang

**Affiliations:** ^1^Respiratory Medicine Unit and National Institute for Health Research (NIHR), Nuffield Department of Medicine Experimental Medicine, Oxford Biomedical Research Centre (BRC), University of Oxford, Oxfordshire, United Kingdom; ^2^Division of Respiratory Medicine, Department of Integrated Traditional Chinese and Western Medicine, West China Hospital, Sichuan University, Chengdu, China

**Keywords:** mucosal-associated invariant T cell, activation, innate, T cells, human, mouse, review

## Abstract

Mucosal associated invariant T (MAIT) cells are striking in their abundance and their strict conservation across 150 million years of mammalian evolution, implying they must fulfill critical immunological function(s). MAIT cells are defined by their expression of a semi-invariant αβ TCR which recognizes biosynthetic derivatives of riboflavin synthesis presented on MR1. Initial studies focused on their role in detecting predominantly intracellular bacterial and mycobacterial infections. However, it is now recognized that there are several modes of MAIT cell activation and these are related to activation of distinct transcriptional programmes, each associated with distinct functional roles. In this minireview, we summarize current knowledge from human and animal studies of MAIT cell activation induced (1) in an MR1-TCR dependent manner in the context of inflammatory danger signals and associated with antibacterial host defense; (2) in an MR1-TCR independent manner by the cytokines interleukin(IL)-12/-15/-18 and type I interferon, which is associated with antiviral responses; and (3) a recently-described TCR-dependent “tissue repair” programme which is associated with accelerated wound healing in the context of commensal microbiota. Because of this capability for diverse functional responses in diverse immunological contexts, these intriguing cells now appear to be multifunctional effectors central to the interface of innate and adaptive immunity.

## Introduction

Mucosal-associated invariant T (MAIT) cells are innate-like T cells defined by their semi-invariant αβ T cell receptor (TCR) which recognizes small-molecule biosynthetic derivatives of riboflavin synthesis (1–3) presented on the restriction molecule major histocompatibility complex (MHC)-related protein-1 (MR1) (4). MAIT cells were described in 1999 (5) based on their TCR comprising a semi-invariant TCR-α chain—usually Vα7.2–Jα33/12/20 in humans, Vα19–Jα33 in mice—predominantly associated with the β-chains Vβ2/Vβ13 in humans and Vβ6/Vβ8 in mice (5, 7).

MAIT cells contrast with conventional T cells which have highly variable TCRs, capable of targeting a vast array of peptide epitopes produced by viruses, bacteria and malignant cells. Conventional T cells therefore have exquisite specificity for individual peptides, and individual clones may undergo massive expansion, to provide T cell memory. However, at the first encounter with a pathogen the frequency of any individual peptide-specific T cell will be very low. In contrast, the MAIT cell TCR provides an innate capacity to respond to a specific set of ligands without the need for expansion.

A key discovery was the identification of these ligands presented on MR1, which include the potent MAIT cell ligands 5-(2-oxopropylideneamino)-6-D-ribitylaminouracil (5-OP-RU) and 5-(2-oxoethylideneamino)-6-D-ribitylaminouracil (5-OE-RU) (2, 8) produced by a wide variety of bacteria, mycobacteria and yeasts during riboflavin (vitamin B2) synthesis (3, 9–12). This pathway is absent from mammals; therefore, its immune detection allows effective host-pathogen discrimination.

Several properties of MAIT cells imply fundamental roles in mammalian immunity. First, MAIT cells have an intrinsic effector-memory phenotype, usually CD45RA^−^CD45RO^+^ CD95^Hi^CD62L^Lo^CD44^Hi^ (4, 13–15), with capacity for rapid secretion of several pro-inflammatory cytokines (13, 15). Second, MAIT cells are remarkably abundant in human tissues, typically comprising 1–4% of all T cells in peripheral blood (16, 17) and up to 10% of airway T cells (18, 19) and 20–40% of liver T cells (13, 20). Moreover, as each TCR recognizes the same ligand, early in an immune response, MAIT cells will markedly exceed the numbers of conventional antigen-specific T cells responding to cognate antigens. Third, the MR1-MAIT cell axis is strikingly conserved across 150 million years of mammalian evolution (21), with ~90% sequence homology for MR1 between mouse and human (21), implying a strong evolutionary pressure maintaining the MAIT cell repertoire. Nonetheless identifying the critical functional role(s) played by these cells has not proved straightforward, perhaps because these cells perform not a single, but several distinct functions. Indeed, several new MAIT cell functions have recently been discovered, representing distinct transcriptional programs which can be triggered via distinct activation pathways.

Here we review both the mechanisms and requirements for MAIT cell activation, and our current understanding of their diverse functional roles, including response to bacterial infections, viral infections and tissue repair. Consequently, these diverse roles suggest the potential to harness MAIT cells in protection against infectious disease, in vaccine design and in promoting wound healing.

## MAIT Cell Activation

### TCR Mediated Activation

MAIT cells can be activated in response to TCR ligation by riboflavin intermediates presented on MR1, under co-stimulatory signals from specific cytokines or toll-like receptors (TLR) (22). Activated cells expand substantially inducing a rapid innate-like immune response and effector functions including anti-microbial cytotoxic products, inflammatory chemokines, and cytokines.

During riboflavin biosynthesis, the pyrimidines 5-OP-RU and 5-OE-RU are generated from the precursor 5-amino-6-D-ribitylaminouracil (5-A-RU) by non-enzymatic condensation with methylglyoxal and glyoxal, respectively (1, 2, 23). RibD is a key gene in this pathway, encoding a pyrimidine deaminase/reductase that generates 5-A-RU. RibD expression correlates with MAIT cell response (24). Thus, bacteria possessing this pathway, for instance, *Escherichia, Lactobacillus, Staphylococcus* (9), *Shigella Flexneri* (25)*, Salmonella, Mycobacteria*, and *Clostridioides* species (3, 9, 26) besides *Mycobacteria* (27) or fungi equipped with riboflavin synthesis, such as *Saccharomyces* (9), *Candida* (9, 28), and *Aspergillus* (29) can activate MAIT cells in an MR1-dependent manner, while other bacterial species lacking the full riboflavin pathway such as *Enterococcus faecalis* (9) and *Listeria monocytogenes* (27) are not.

Unlike conventional T cells which recognize peptide antigen presented by MHC molecules, MAIT cells are restricted by MR1, a non-polymorphic, β2-microglobulin-associated antigen-presenting molecule, widely expressed in multiple tissues (30, 31). Unlike class 1 and class 2 MHC, MR1 does not constitutively present self-ligands. Generally, MR1 molecules reside in the endoplasmic reticulum (ER) in an incompletely folded ligand-receptive conformation, free of β2 microglobulin. Riboflavin metabolites are transported to the ER, bind MR1 via formation of a Schiff base, followed by a complete folding and association with β2-microglobulin. The ternary complex then traffics through the ER and the Golgi to the cell membrane (32). Although recycling of the MR1 molecule can occur (33), most MR1 molecules are degraded and reinternalized intracellularly, which contributes to MR1's rapid presentation of extracellular riboflavin antigens and MAIT cells' rapid activation (32). Moreover, NF-κB signaling is necessary for MR1 signal transduction (34). Either bone marrow-derived antigen-presenting cell (APC) such as dendritic cells (27, 35), monocytes (9, 13), macrophages (35), B cells (36, 37), or non-bone marrow-derived epithelial cells (27, 37) can activate MAIT cells via MR1 (38).

As with conventional T cells, MR1-TCR signaling alone is insufficient to fully activate MAIT cells which also require co-stimulation (22, 38–40) by CD28 (39), TLR agonists, bacterial products or cytokines (22). Such cytokines include interleukin (IL)-7 (41, 42), tumor necrosis factor (TNF) (43), type-I interferons (IFNs) (44), IL-1β and/or IL-23 (38, 41). MAIT cells express several cytokine receptors including IL-7R, IL-12R, IL-15R, IL-18R, and IL-23R (9, 13, 38). IL-7 enhances MAIT cell responses to bacteria and promotes cytotoxicity (42). IL-12 and IL-18 potentiate MR1-dependent bacterial MAIT cell activation (34, 45). Agonists of the pathogen recognition receptors TLR1, TLR2 and TLR6 in humans (34), and TLR3, TLR4, TLR6/2, and TLR9 in mice (22, 46) promote MAIT cell activation mainly in an indirect way through the activation of APCs via enhancement of MR1 presentation, stimulation of cytotoxic molecules and inflammatory cytokines or up-regulation of co-stimulatory ligands (22, 34, 47, 48). In addition, inducible T cell co-stimulator (ICOS), highly expressed by MAIT cells is also essential for optimal activation and maintenance of retinoic acid-related orphan receptor γt (RORγt) expression (38).

Whilst the MAIT cell TCR-α chain is usually Vα7.2–Jα33/12/20 in humans, the β chain is more diverse: Vβ2 and Vβ13 are the most common. Some data suggest certain Vβ segments are associated with decreased TCR-dependent MAIT cell responses (49). Recently non-cognate TCR-dependent MAIT cell activation has been described for several bacterial superantigens. Although group A streptococci lack the riboflavin pathway, three molecules they produce bind specifically to Vβ2 leading to rapid TNF-dominated MAIT cell activation, likely contributing to the cytokine storm in streptococcal toxic shock syndrome (50). Likewise, in toxic shock induced by *S. aureus*, the staphylococcal enterotoxin B acts as a superantigen preferentially ligating MAIT cell Vβ13.2 with MHCII (51).

### TCR-Independent Activation

In the absence of TCR-mediated antigen recognition, MAIT cells can also be partially activated by cytokines, such as IL-7, IL-12, IL-15, IL-18, and type-I IFNs, broadening the potential range of pathogens to which MAIT cells can respond to include viruses (35, 52–54). IL-7 can induce expression of cytolytic effector molecules (42). IL-12 or IL-15 together with IL-18, produced from APCs in response to TLR ligands, can directly stimulate MAIT cells to produce IFN-γ (35, 48, 55) and release granzyme B and perforin (45). IFN-α/β alone can activate MAIT cells but not induce cytokine production or upregulation of co-stimulatory molecules (44). Type-I IFNs induce significant IFN-γ and granzyme B only when combined with IL-12 or IL-18 (35). Likewise, the gut-associated pro-inflammatory cytokine, TNF-like protein 1A (TL1A/TNFSF15) activate MAIT cells in combination with IL-12 and IL-18 (56).

**Figure 1:**
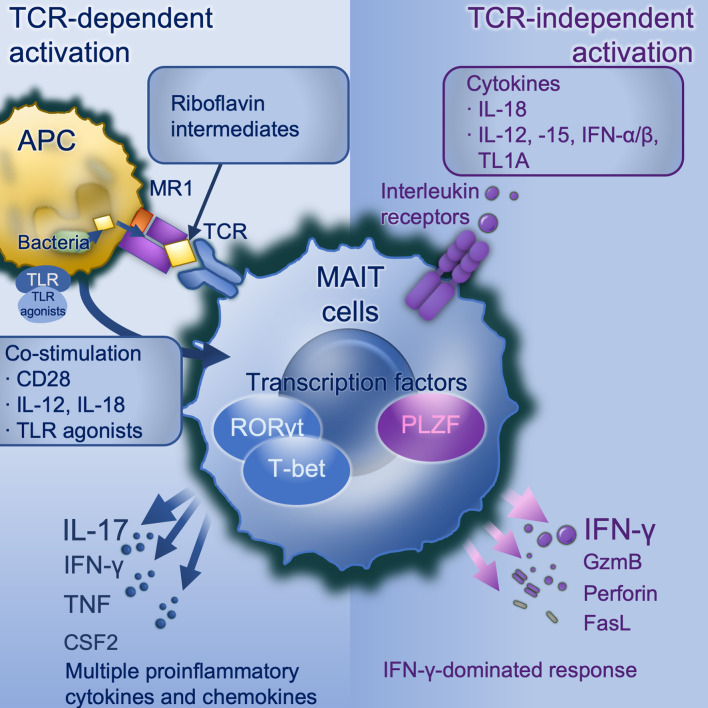
MAIT cell activation. TCR-dependent activation requires microbially-derived riboflavin intermediates such as 5-OP-RU to be presented on MR1 to a MAIT cell TCR in conjunction with co-stimulation. Co-stimulation may include CD28 and/or cytokines particularly IL-12/-18, and can be induced by danger signals including TLR agonists (in mice TLRs 3, 4, 6/2, or 9, in human TLRs 1, 2, or 6). TCR dependent activation induces a strong and broad production of pro-inflammatory cytokines and chemokines, dominated by IL-17A under the control of the transcription factor RORγt. TCR-independent activation is driven by IL-18 in synergy with IL-12, or−15, or IFN-α or -β, and potentiated by TL1A. This activates a more modest cytokine response dominated by IFN-γ under the control of PLZF. APC, antigen-presenting cell; CD, cluster of differentiation; CSF2, colony stimulating factor 2 (GM-CSF); GzmB, granzyme B; IFN, interferon; IL, interleukin; MAIT, mucosal associated invariant T; MR1, major histocompatibility complex-related protein-1; PLZF, Promyelocytic leukaemia zinc finger protein; RORγt, retinoic acid-related orphan receptor gamma; TCR, T cell receptor; TL1A, tumor necrosis factor (TNF)-like protein 1A (TNFSF15); TLR, toll-like receptor.

TCR-mediated and -independent activation work synergistically in optimal MAIT cell activation (Figure 1). Upon stimulation, there is increased expression of activation markers CD69 and CD25, degranulation marker CD107a (9, 25, 57), production of cytotoxic substances such as perforin and granzyme B (13, 42, 58), secretion of pro-inflammatory cytokines including IFN-γ, TNF, IL-17, and colony stimulating factor 2 (CSF2/GM-CSF) (9, 12, 13) and release of chemokines such as XCL1, CCL3, CCL4, and CXCL16 (40, 46). MAIT cells exert antimicrobial activity not only by direct recognition and killing of infected cells, but also indirectly, for example by recruiting neutrophils (59), increasing bactericidal activity of phagocytes (60), promoting the production of IFN-γ from DCs (35), and promoting monocyte to DC differentiation (61). Despite the considerable overlap in transcriptional and functional profiles, there are differences between these two modes of activation. TCR stimulation generally results in a more rapid immune response and multiple pro-inflammatory cytokines and chemokines production than cytokine stimulation alone. Elevated expression of RORγt, and the cytokines IL-17A, TNF, and CSF2 are seen with TCR-mediated activation, consistent with a Tc17-like phenotype (46, 59, 62), whilst TCR-independent activation is dominated by IFN-γ, perforin and granzyme B under the control of promyelocytic leukemia zinc finger (PLZF) (46) and T-bet (59), consistent with a Tc1-like phenotype. Importantly it is TCR-dependent activation which induces a tissue-repair programme (46, 56, 59). Knowing TCR-dependent and -independent MAIT cell responses are distinct, there remain many unanswered questions. For example, do MAIT cells shift between Tc17-like phenotype and Tc1-like phenotype? If such plasticity exists, what conditions are required and what are the influences?

## MAIT Cell Functions in Infection

As a specific population of innate-like lymphoid cells, MAIT cells are involved in early immunity against infection in peripheral tissue, with a more rapid response to pathogens and shorter time to effector function *in vivo* than conventional MHC-restricted T cells in infectious disease (63). Next, we review MAIT cell functions antibacterial and antiviral host defense. The pathogens, activation elements, and effectors responses are summarized in Supplementary Table 1.

### Antibacterial Host Defense

**Figure 2:**
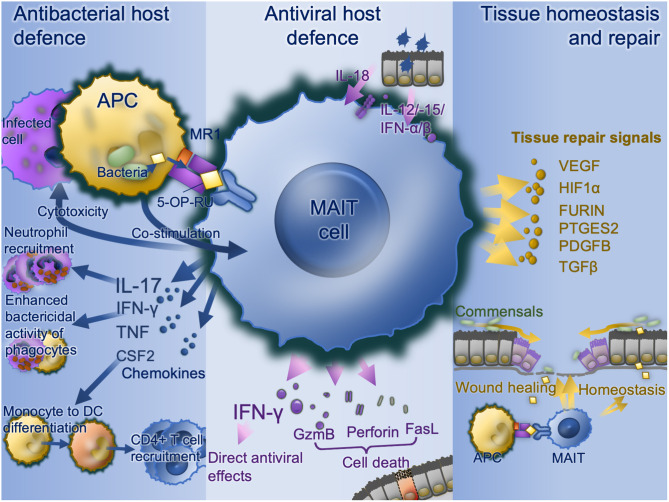
MAIT cell functions. Bacterially-infected cells present MAIT cell ligands on MR1 with co-stimulation and activate MAIT cell antibacterial defense functions including IFN-γ enhancement of antibacterial function in phagocytes, recruitment of neutrophils via IL-17 and differentiation of inflammatory monocytes to DCs in turn driving CD4 T cell recruitment. Virally infected cells produce IL-18, IL-12, IL-15, and type I interferons which upregulates IFN-γ with direct antiviral effects as well as direct cytotoxic MAIT cell functions via granzyme B, perforin, and Fas ligand. Commensal derived MAIT cell ligands, in the absence of co-stimulation stimulate MAIT cells to promote tissue homeostasis. Where a mucosal barrier is damaged these signals are enhanced and accompanied by damage signals to drive accelerated tissue repair signals. APC, antigen-presenting cell; CSF2, colony stimulating factor 2 (GM-CSF); DC, dendritic cell; FasL, Fas cell surface death receptor ligand; GzmB, granzyme B; IFN, interferon; IL, interleukin; MAIT, mucosal associated invariant T; MR1, major histocompatibility complex-related protein-1; TCR, T cell receptor.

A wide range of bacterial, mycobacterial, and fungal pathogens have been shown to activate MAIT cells *in vitro* (Figure 2). These pathogens all express the riboflavin pathway and activation is via TCR-dependent activation. MAIT cells co-cultured with bacterially-infected monocytes (9, 13, 35, 51) or *M. tuberculosis-*infected lung epithelial cell lines (27) release IFN-γ in an MR-dependent manner. MAIT cell TCR-transgenic mice were better protected against infection by *E. coli* or *M. abscessus* than Mr1^−/−^ MAIT cell TCR-transgenic mice (9). Furthermore, bacterially-activated MAIT cells express perforin, undergo degranulation and can directly kill *E. coli-*infected human epithelial cell lines (HeLa-MR1) modified to over-express MR1 (25). With the intracellularly-invasive *Shigella*, this cell killing may occur in parental HeLa cells, suggesting a predilection for MAIT cells to respond to intracellular bacteria (25). Thus, there is potent inhibition of *Mycobacterium bovis* BCG growth within macrophages when co-cultured with MAIT cells (12). Consistent with this, *Streptococcus pneumoniae* has a poor capacity to activate MAIT cells when the APC is a monocyte or monocyte cell line, and this activation is dependent on cytokines rather than MR1 (64), perhaps because of monocytes' poor phagocytic and antigen-presenting capacity (65, 66). Conversely, *S. pneumoniae* does induce MR1-dependent activation in the presence of monocyte-derived macrophages, which have a greater phagocytic capacity (64). Likewise, *S. pneumoniae* activates MAIT cells in the presence of human monocyte-derived dendritic cells, in an MR1-dependent manner, with the extent of activation correlating with the activity of the RibD operon (24). In co-culture, MAIT cells reduce growth of *S. pneumoniae* within infected primary bronchial epithelial cells.

To date, few pathogens have been found to induce MAIT cell expansion *in vivo*, and interestingly those that have are also predominantly intracellular pathogens. In a murine model, using a live vaccine strain (LVS) of the rare opportunistic intracellular pathogen *Francisella tularensis*, pulmonary MAIT cells expand markedly during acute intranasal infection (67). These cells express IFN-γ, IL-17, TNF, and inducible nitric oxide synthase (67) as well as GM-CSF which enhances pulmonary recruitment of inflammatory monocytes (61). There is impaired protection against infection by pulmonary *F. tularensis* LVS in CD4^+^CD8^+^-depleted MR1^−/−^ mice in contrast to their wild-type counterpart, which demonstrates MAIT cells' significance in mucosal immunity in absence of CD4^+^ and CD8^+^ T cells. In a second model of intracellular pulmonary bacterial infection, intranasal *S*. Typhimurium induced rapid, MR1-dependent expansion of pulmonary MAIT cells frequencies which persisted as an expanded population long-term comprising 35–50% of all pulmonary αβ T cells (22).

In the *S*. Typhimurium model, bacterial clearance was not dependent on MAIT cells, due to other redundant mechanisms including conventional CD4^+^ and CD8^+^ T cells (22). Impaired control of microbial growth *in vivo* has been observed with *F. tularensis* (61, 67) and the mycobacteria *M. abscessus* (9) and *M. bovis* BCG (12) in MR1^−/−^ mice which have an absolute deficiency of MAIT cells. In a murine model of Legionnaire's disease, two clinically-important strains of *Legionella—pneumophila* and *longbeachae—*activated human MAIT cells *in vitro* (62). *L. longbeachae* induced rapid pulmonary MAIT cell expansion, by up to 580-fold over 7 days, which persisted long-term, with a similar effect observed with *L. pneumophila*. 5-bromo-2′-deoxyuridine incorporation showed this was due to early, local MAIT cell proliferation in the lung and draining lymph node. MR1^−/−^ mice had delayed bacterial clearance at days 10–14 post-infection, although in both models this effect was small, due to immunological redundancy. To remove these additional layers of immunological redundancy, we adoptively transferred MR1-5-OP-RU positive MAIT cells into Rag2^−/−^γC^−/−^ mice which lack T, B and NK cells. MAIT cells were sufficient to rescue mice completely from fatal *L. longbeachae* infection (62).

How do MAIT cells provide this protection? Using adoptive transfer from mice deficient in various signaling pathways, we showed this immune protection depended predominantly on MAIT cell IFN-γ, and to a lesser extent on GM-CSF, but not, with this organism, on perforin or granzyme (62). *Legionella* infects inflammatory cells, particularly neutrophils and macrophages, rather than epithelia and it is likely MAIT-derived IFN-γ limits intracellular growth of bacteria in these cells through multiple mechanisms including enhancement of oxidative burst, nitric oxide production, antigen presentation, phagocytosis and upregulation of CD80/86 co-stimulation, cytokines and chemokines (68). IFN-γ is also critical for protection against mycobacterial disease including *M. tuberculosis*, so this early production of MAIT cell-derived IFN-γ may likely be an important and non-redundant component of protection against mycobacteria. Indeed, polymorphism in MR1 is associated with susceptibility to *M. tuberculosis* (69).

Might MAIT cells be important in more common respiratory diseases? Persistent infection with *Haemophilus influenzae*, which can persist intracellularly (70), can drive inflammation in chronic obstructive pulmonary disease (COPD) (71) and severe neutrophilic asthma (72, 73) and enhance susceptibility to virally-induced inflammation (74, 75). MAIT cells are abundant in the normal airway mucosa, but we have shown frequencies are markedly reduced by therapeutic corticosteroids in both asthma (18) and COPD (19). *In vitro H. influenzae* enhances surface expression of MR1 on human pulmonary macrophages and induces MAIT cell IFN-γ, but both functions are also inhibited by corticosteroids, which may explain the enhanced susceptibility to bacterial pneumonias in airways diseases (19), suggesting a potential clinical benefit from more judicious use of inhaled steroids or MAIT-cell enhancing strategies.

MAIT cells are therefore capable of providing TCR-dependent rapid immune defense against a range of pathogenic intracellular bacteria. However, paradoxically, despite evidence of strong evolutionary pressure to maintain MAIT cell populations, it has been hard to identify a profound, non-redundant phenotype of pure MAIT cell deficiency either in the clinic or in animal models. The explanation may be that in addition to this undoubtedly important antibacterial role, it is now clear that MAIT cells perform additional roles in mucosal immunology, including antiviral host defense and tissue repair.

### Antiviral Host Defense

Conventional T cells are critical for eventual clearance of most viruses by producing a peptide-specific cell-mediated immune response, which takes 4–7 days to evolve. Given their capacity for cytokine-mediated, TCR-independent activation, do MAIT cells play a role in anti-viral host-defense? Virus-induced activation of CD161^+^Vα7.2^+^ MAIT cells was observed *in vivo* in human peripheral blood MAIT cells during clinical infection of dengue, hepatitis C and influenza A (35), as well as at the peak viremia stage during acute infection by human immunodeficiency virus (76). Activation, measured by CD38 or granzyme B upregulation, increased during each infection *in vivo* whilst also *in vitro* MAIT cells upregulated CD69 and IFN-γ in the presence of virally-infected APCs. Similar findings were observed in a separate report of patients hospitalized with the H7N9 strain of influenza A virus, and interestingly higher MAIT cell frequencies were associated with subsequent recovery (53). Indeed cytokine-activated MAIT cells could reduce replication of hepatitis C *in vitro* in an IFN-γ dependent manner (35). However, the correlative clinical observations cannot prove causality, and neither study addressed whether the consequences of virus-induced MAIT cell activation in the intact host would be a beneficial contribution to antiviral defense, or conversely a worsening of immune-pathology, perhaps even augmentation of the cytokine-storm which can prove fatal in acute influenza infection.

To address this question we compared survival in mice infected with H1N1 influenza A virus in the presence or absence of an intact MR1: MAIT cell axis (52). MR1-tetramer^+^ MAIT cells accumulated in the lungs and were activated, with MAIT cell frequencies and CD25, CD69, and granzyme B upregulation peaking at day 5 post-infection, at least 48 h earlier than peak frequencies of conventional CD8^+^ T cells (defined as TCRβ^+^ CD45.2^+^ CD19^−^ MR1-5-OP-RU tetramer^−^ CD8^+^cells). Activation was dependent predominantly on IL-12 and to a lesser extent on IL-15, IL-18, and IFN-α. MAIT cell-deficient MR1^−/−^ mice showed enhanced weight loss and mortality which was ameliorated by prior adoptive transfer of pulmonary MAIT cells in both immunocompetent and immunodeficient Rag2^−/−^γC^−/−^ mice. This confirmed that MAIT cells did not worsen immunopathology, but rather they contributed to immune protection. Moreover, current yearly influenza vaccines show limited efficacy and little heterologous protection between strains, whereas the TCR-independent nature of the antiviral MAIT cell response and the adoptive transfer experiments suggest the possibility of clinical benefit from therapeutic enhancement of airway MAIT cell numbers, such as a pre-exposure boosting using inhaled MAIT cell ligands. Such an approach would require clinical trials.

## MAIT Cell Function in Tissue Repair and Homeostasis

Recently a third, distinct function has been discovered for MAIT cells. Using a transcriptomic approach on sorted MR1-tetramer^+^ cells, we sought to define the transcriptome of an activated MAIT cell in both human and mice. In addition to the strong expression of pro-inflammatory cytokines, we discovered in both species TCR-mediated activation-induced expression of a tissue-repair programme (46). The genes upregulated in both species included TNF, CSF2, HIF1A, FURIN, VEGFB, PTGES2, PDGFB, TGFB1, MMP25, and HMGB1. These genes had previously been identified as a geneset expressed by skin-homing Tc17 cells induced by commensal flora and able to accelerate repair of an epithelial wound in mice (77). Such Tc17 cells were restricted by another MHC class 1b molecule H2-M3, but this molecule is absent in humans and given their commensal dependence and capacity for IL-17 production it seemed likely MAIT cells might share this programme. Indeed in a comparative transcriptomic analysis of different T cell subsets in the ImmGen database (78) activated MAIT cells shared the greatest similarity with these commensal-induced epithelial Tc17 cells (46). This tissue repair programme is observed in MAIT cells stimulated by TCR ligands but not by cytokine-mediated stimulation alone (56, 59). Supernatants from TCR-activated MAIT cells accelerated wound closure in an intestinal epithelial cell line system (56).

Further studies *in vivo* have shown that skin colonization of germ-free mice with a common skin-commensal *Staphylococcus epidermidis* induced expansion of cutaneous MAIT cell populations and upregulation of this tissue repair programme in cutaneous MAIT cells (79). These MAIT cells are predominantly localized in the dermis near the dermal-epidermal junction and their MAIT cell expansion depended on 5-OP-RU and MR1. Moreover, these MAIT cells could accelerate the closure of a punch-biopsy induced skin wound, which was enhanced by application of topical 5-OP-RU.

Commensal organisms play a fundamental role in the development, function and homeostasis of the host immune system. Maintenance of the optimal symbiotic relationship between commensal microbiota and the immune system allows protective immune responses to occasional invasive pathogens (80, 81). Since MAIT cells are predominantly located in tissues colonized by commensal microbes, with broad antimicrobial specificity and tissue-repair function, MAIT cells' response to commensal organisms and tissue damage and infections may be as important for the restoration of homeostasis as their role in protection against pathogen invasion. Furthermore, it seems likely that, depending on the distinct tissue microenvironment, MAIT cells may express both antibacterial and also tissue repair functions at different stages in the evolution of an infectious or physical injury.

## MAIT Cell Function in Autoimmunity and Inflammation

Conventional T cells are implicated as effectors in many organ-specific autoimmune diseases such as type-1 diabetes or multiple sclerosis, but strong HLA associations in a range of systemic autoimmune diseases imply a pathogenic role in these diseases as well. What role might MAIT cells play in these diseases? As the MAIT cell ligands are not synthesized by human cells, MAIT cell activation in these conditions would be presumed to be via the cytokine-mediated TCR-independent pathway. Changes in MAIT cell frequencies and phenotype are observed in a range of autoimmune conditions.

Blood MAIT cells are decreased in children with type 1 diabetes mellitus. In the non-obese diabetic mouse MR1^−/−^ mice have accelerated diabetes and increased gut permeability, suggesting MAIT cells may be protective against diabetes by supporting intestinal mucosal integrity, although the data are complicated by evidence in mice and humans of MAIT cell activation, exhaustion and capacity for islet-cell killing (82). Similarly, in type 2 diabetes peripheral blood MAIT cells are reduced in frequency, associated with increased caspase-3-dependent apoptosis (83).

In rheumatoid arthritis MAIT cell frequencies are increased in synovial tissue and so may contribute to maturation and cross-differentiation of T cells locally (84). Consistent with a pathogenic role, inflammation is reduced in murine collagen-induced arthritis in MR1^−/−^ mice (85).

MAIT cells are increased in inflammatory lesions in human multiple sclerosis (86), although data from animal models suggest they may play a protective role in the lesions as inflammation and pathology are reduced by adoptive transfer and exaggerated in MR1^−/−^ animals (87). Likewise, inflammation is suppressed by inhibitory MAIT cell ligands in an animal model of systemic lupus erythematosus (88).

In the gut, MAIT cells are found in proximity to *Helicobacter pylori* in human gastric mucosa, and in mice, MAIT cells were associated with accelerated *H. pylori* gastritis (89). In inflammatory bowel disease, several studies show decreases in peripheral blood MAIT cell frequencies and generally an increase in intestinal tissue (90–93), with increased IL-17 and IL-22 production by blood MAIT cells, although it remains to be seen if these changes are causally linked and whether they are pathogenic or protective by restoring mucosal integrity (94, 95).

## Potential Clinical Translation of MAIT Cell Biology

Several characteristics of MAIT cells—their abundancy, their intrinsic effector memory phenotype (13), their mucosal distribution and the invariant nature of their receptor—make them excellent candidates to harness in the development of vaccines. In a therapeutic vaccine, for instance, for a respiratory infection, MAIT cell frequencies might be rapidly increased by stimulation with a MAIT cell ligand and co-stimulation, as has been shown in mice (22, 46, 62). More likely if more stable MAIT cell-activating ligands could be developed, these could be added to B cell or T cell prophylactic vaccines to enhance biological adjuvancy (96).

In autoimmune diseases in which MAIT cells had a predominantly pathological role, it would be possible to inhibit MAIT cells in a highly-selective manner using potent, inhibitory MAIT cell ligands such as acetyl-6-formylpterin (97). As orally-active small molecules, these could be attractive targets.

These cells' recently discovered role in promoting wound healing is perhaps the most exciting, from a translational perspective. Healing can be slow in chronic skin wounds such as leg ulcers, sacral pressure sores or burns, and could potentially be accelerated either by local wound re-colonization with riboflavin-synthesizing commensals, or through topical application of synthetic MAIT cell ligands. The latter approach has already been tested in a proof-of-principle murine study (79), and could very easily be translated into large-scale clinical trials.

In summary, the conservation and abundance of MAIT cells is likely explained by their broad range of functionality attributable to different modes of activation, each triggering a distinct transcriptomic programme. Because of their capability for diverse functional responses in diverse immunological contexts, these intriguing cells now appear to be multifunctional effectors central to the interface of innate and adaptive immunity. Already three major functions—antibacterial host defense, antiviral host defense, tissue repair and homeostasis—have been described for these intriguing cells, but it is likely other functions remain to be discovered.

## Author Contributions

TH and X-WZ jointly conceived the article, conducted the literature review, and drafted the manuscript. All authors approved the final manuscript.

## Conflict of Interest

The authors declare that the research was conducted in the absence of any commercial or financial relationships that could be construed as a potential conflict of interest. The handling editor declared a past collaboration with one of the authors TH.

## Supplementary Material

Supplementary Table 1 Effects on MAIT cell activation and functions of various bacterial pathogens.

## Abbreviations

5-OE-RU: 5-(2-oxoethylideneamino)-6-D-ribitylaminouracil
5-OP-RU: 5-(2-oxopropylideneamino)-6-D-ribitylaminouracil
MAIT: mucosal associated invariant T
MHC: major histocompatibility complex
MR1: major histocompatibility complex-related protein-1
TCR: T cell receptor.
